# The global prevalence of parasites in non-biting flies as vectors: a systematic review and meta-analysis

**DOI:** 10.1186/s13071-023-05650-2

**Published:** 2023-01-23

**Authors:** Yufeng Liu, Yuancai Chen, Nanhao Wang, Huikai Qin, Longxian Zhang, Sumei Zhang

**Affiliations:** 1 College of Veterinary Medicine, Henan Agricultural University, Zhengzhou, 450046 People’s Republic of China; 2 International Joint Research Center for Animal Immunology of China, Zhengzhou, Henan People’s Republic of China; 3 Key Laboratory of Quality and Safety Control of Poultry Products (Zhengzhou), Ministry of Agriculture and Rural Affairs, Zhengzhou, People’s Republic of China

**Keywords:** Non-biting flies, Vectors, Parasites, Meta-analysis

## Abstract

**Background:**

Non-biting flies such as the house fly (*Musca domestica*), the Australian sheep blowfly (*Lucilia cuprina*) and the oriental latrine fly (*Chrysomya megacephala*) may carry many parasites. In the present study, we performed a systematic overview of the different species of parasites carried by non-biting flies, as well as of isolation methods, different geographical distribution, seasonality and risk assessment.

**Methods:**

A meta-analysis was carried out with the aim to review the global prevalence of parasite transmission in non-biting flies. A total sample size of 28,718 non-biting flies reported in studies worldwide satisfied the predetermined selection criteria and was included in the quantitative analysis.

**Results:**

The global prevalence of parasites in non-biting flies was 42.5% (95% confidence interval [CI] 31.9–53.2%;* n* = 15,888/28,718), with the highest prevalence found for non-biting flies in Africa (58.3%; 95% CI 47.4–69.3%;* n* = 9144/13,366). A total of 43% (95% CI 32.1–54.4%;* n* = 7234/15,282) of house flies (*M. domestica*), the fly species considered to be the most closely associated with humans and animals, were found with parasites. The prevalence of parasites in the intestine of non-biting flies was 37.1% (95% CI 22.7–51.5%; * n* = 1045/3817), which was significantly higher than the prevalence of parasites isolated from the body surface (35.1%; 95% CI 20.8–49.4%;* n* = 1199/3649; *P* < 0.01). Of the 27 reported parasites, a total of 20 known zoonotic parasites were identified, with an infection rate of 38.1% (95% CI 28.2–48.0%;* n* = 13,572/28,494).

**Conclusions:**

This study provides a theoretical basis for the public health and ecological significance of parasites transmitted by non-biting flies.

**Graphical Abstract:**

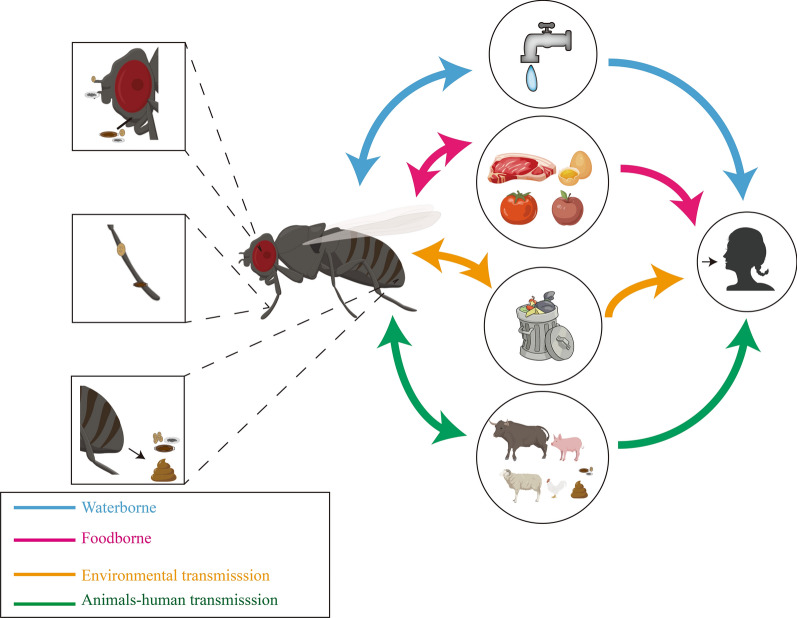

**Supplementary Information:**

The online version contains supplementary material available at 10.1186/s13071-023-05650-2.

## Background

Many species of flies (Diptera) are closely associated with humans and can complete their entire life-cycle in the close proximity of human habitations and areas with domestic animals [1]. At least 305 fly species belonging to the Muscidae, Calliphoridae and Sarcophagidae families are known to transmit diseases [2]. Flies can be divided into non-biting flies and biting flies. Non-biting flies are often commonly found in areas of human and animal activities, such as food markets, restaurants and poultry and livestock farms where they have the potential to be vectors of diseases [3, 4].

Non-biting flies can have sponging mouthparts, which are mainly used to lick and suck food, and can be either mechanical or biological vectors of food-borne pathogens [5, 6]. Biting flies have piercing-sucking mouthparts and suck blood through punctures made by piercing human and animal skin [7, 8]. Non-biting flies are mechanical carriers of pathogens that adhere to their body, mouthparts and body hair. These flies transmit pathogens mainly by contact, and they can contaminate their surrounding environment as they move about [9]. Food-borne pathogens can reproduce in the intestines of non-biting flies and infect food through fly excretion and regurgitation [10]. After exposure to food-borne pathogens, humans and animals can experience diarrhea and other symptoms that sometimes lead to death [11]. As such, non-biting flies can carry many pathogens, and these pathogens have detrimental effects on public health.

Non-biting flies can carry more than 100 kinds of pathogens, including parasites, bacteria, fungi and viruses [12]. Among these, parasite eggs/cysts (such as those of *Cryptosporidium* spp., *Giardia spp.*, *Taenia* spp.) have been isolated from the body surface and intestines of non-biting flies, with most being zoonotic parasites. Such parasites harm humans and animals by depriving the host of nutrients and damaging host tissues and organs [13, 14].

No systematic investigation has been conducted on the parasites carried by non-biting flies. This study provides a systematic overview of the different species, isolation methods, different geographical distribution, seasonality and risk assessment of parasites carried by non-biting flies.

## Methods

### Search strategy

**Fig. 1:**
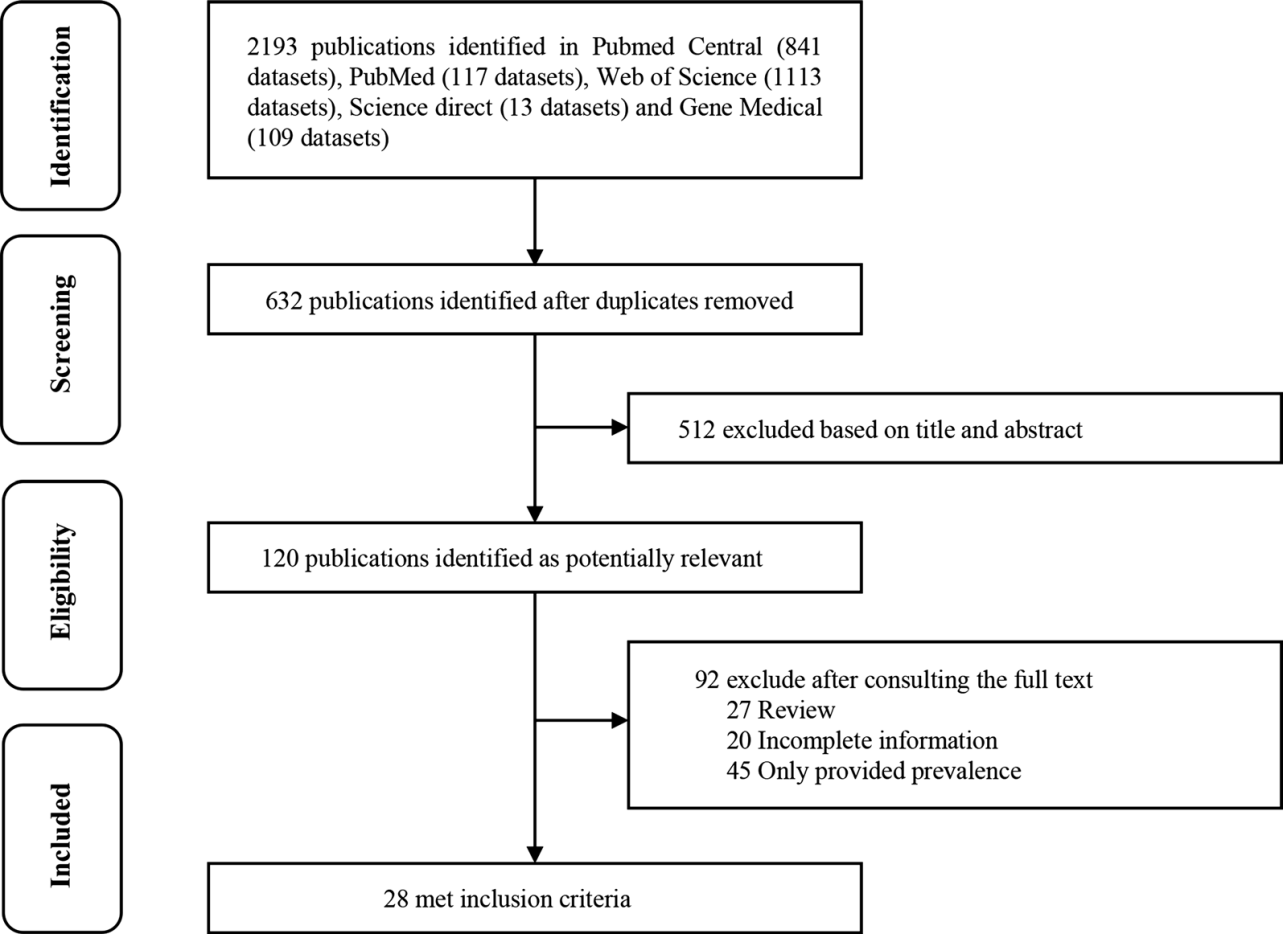
PRISMA flow diagram on the different stages of the literature search process

To determine the prevalence of parasites transmitted by non-biting flies worldwide, we performed a systematic search of the PubMed, PubMed Central, GeenMedical, Web of Science and Science Direct electronic databases, with the aim to identify relevant literature (Fig. 1). The search was performed according to the Preferred Reporting Items of Systematic Reviews and Meta-Analyses (PRISMA) guidelines [15]. The keywords used were: “house fly (*Musca domestica*),” “*Lucilia sericata*,” “*Chrysomya megacephala*,” “*Ascaris lumbricoides*,” “*Trichuris trichiura*,” “*Taenia solium*,” “*Entamoeba coli*,” “*Enterobius vermicularis*,” “Hookworm,” “*Strongyloides stercoralis*,” “*Hymenolepis nana*,” “*Entamoeba histolytica*,” “*Cryptosporidium parvum*,” “*Giardia lamblia*” and “*Enterocytozoon bieneusi*”, using “AND” and/or “OR” Boolean operators [16]. The search formula used was (host 1) OR (host 2) AND (parasite 1) OR (parasite 2). . Literature data were obtained based on different non-biting fly species, different parasite species, different national geographic distribution, seasonality, sample size, positive number and identification method [17].

### Selection criteria

We searched all English articles on the epidemiology of parasites carried by non-biting flies without a publishing time limit. According to the PRISMA guidelines (Additional file 1: Table S1), titles were screened first for eligibility based on full and legible citations and journal article titles only [18]. Then, in groups of two reviewers at a time, the titles and abstracts were assessed. Articles meeting the selection criteria include the following points: (i) detailed and comprehensive sample information; (ii) details on sample size and number of positive specimens provided; (iii) peer-reviewed journal articles; and (iv) clear description of detection methods provided.

### Study selection

Articles that did not meet the selection criteria were removed, and articles from which reference data could be extracted were filtered out. Exclusion criteria were: (i) duplicate articles in the five databases; (ii) incomplete information on the sample; (iii) review article; (iv) only the prevalence was provided, without information on sample size and positivity; (v) no details on the sample, and the data is not easy to distinguish; and (vi) experimental studies, letters and articles published in a language other than English (Additional file 1).

### Quality assessment

The Grading of Recommendations Assessment, Development and Evaluation (GRADE) system was used to grade article quality. Article quality was assessed according to current standards, and scores were determined [19]. Each criterion was graded with a score of 1 point. The scoring criteria included whether the study subjects were clearly defined; whether there was ≥ 3 types of grouping analysis; whether the identification method was clearly described; whether the sampling time was reported in sufficient detail; and whether the sample size was > 200. Score grades were divided into 3 grades, with 0–1 indicating low quality, 2–3 indicating medium quality and 4–5 indicating high quality [20].

### Data extraction

All titles, abstracts and full texts were separately screened by two authors (YFL and YCC), and the data were independently extracted. Disagreements were resolved by discussion with a third author (NHW). The data included species of non-biting flies, different parasite species, country, seasonality, total sample sizes, positive sample sizes, identification method, publication year, first author’s name, sampling time and external surface/internal organs of non-biting flies (Table 1).

**Table 1:** Study quality assessment and score grading of the 28 studies included in the meta-analysis

No	First author	Country	Sampling time	No. tested	No. positive	Identification method	Fly species	Parasites^a^	Research object clearly or not	Three or more group analyses or not	Sampled method described in detail or not	Sampled time clearly reported or not	The number of samples is ≥ 200 or not	Score grade	Study quality
1	Clavel et al. [21]	Spain	July 1998–August 1998	600	110	PCR	*Musca domestica*	*C. parvum*	Y	Y	Y	Y	Y	5	High
2	Förster et al. [22]	Germany	May 2007–September 2007	224	68	Microscopy	*Musca domestica*	*A. suum* *M. apri* *S. ransomi* *T. suis*	Y	Y	Y	Y	Y	5	High
3	Doiz et al. [23]	Spain	July 1998–August 1998	600	130	PCR	*Musca domestica*	*G. lamblia*	Y	Y	Y	Y	Y	5	High
4	Roberts et al. [24]	England	–	107	68	Microscopy	*Musca domestica*	*E. histolytica*	Y	N	Y	N	N	2	Medium
5	Pegg et al. [25]	England	–	500	331	Microscopy	*Musca domestica*	*T. canis*	Y	N	Y	N	Y	3	Medium
6	Paliy et al. [26]	Ukraine	–	312	6	Microscopy	*Musca domestica, Muscina stabulans,* *Stomoxys calcitrans*	*T. canis* *T. vulpis* *A. caninum*	Y	N	Y	N	Y	3	Medium
7	Lalander et al. [27]	Sweden	–	224	13	Microscopy	*Aldrichina grahami*	*A. suum*	Y	N	Y	N	Y	3	Medium
8	Ahmadu et al. [28]	Nigeria	July 2014–December 2014	400	254	Microscopy	*Musca domestica*	*E. histolytica* *G. lamblia* *T. solium* *A. lumbricoides* *T. trichiura* *H. nana*	Y	Y	Y	Y	Y	5	High
9	Fetene et al. [29]	Ethiopia	October 2006–June 2007	1306	1089	Microscopy	*Musca domestica, * *Chrysomya rufifacies, * *Musca sorbens, * *Lucilia cuprina, * *Calliphora vicina, * *Chrysomya bezziana, * *Wohlfahrtia magnifica*, *Lucilia sericata*	*A. lumbricoides* *T. trichiura* *Hookworm* *S. stercoralis* *H. nana* *E. histolytica* *G. lamblia* *E. coli* *Cryptosporidium *spp.	Y	Y	Y	Y	Y	5	High
10	Adenusi et al. [30]	Nigeria	November 2011	719	541	Microscopy	*Musca domestica*	*A. lumbricoides* *E. vermicularis* *H. nana* *Hookworm* *S. stercoralis* *T. solium* *T. trichiura* *E. histolytica* *E. coli* *Cryptosporidium *spp.	Y	Y	Y	Y	Y	5	High
11	Getachew et al. [31]	Ethiopia	January 2004–June 2004	9550	6685	Microscopy	*Musca domestica, * *Chrysomya rufifacies*, *Musca sorbens*, *Lucilia cuprina, * *Calliphora vicina, * *Wohlfahrtia magnifica*	*A. lumbricoides* *T. trichiura* *Hookworm* *H. nana* *S. stercoralis* *E. histolytica* *G. lamblia* *E. coli* *Cryptosporidium *spp.	Y	Y	Y	Y	Y	5	High
12	Oyeyemi et al. [32]	Nigeria	January 2014–February 2014	150	67	Microscopy	*Musca domestica*	*A. lumbricoides* *E. vermicularis* *Hookworm* *E. histolytica* *T. trichiura* *T. solium*	Y	Y	Y	Y	N	4	High
13	Adenusi et al. [33]	Nigeria	October 2009–March 2010	303	185	Microscopy	*Musca domestica, Chrysomya megacephala* *Musca sorbens*	*A. lumbricoides* *T. trichiura* *T. solium*	Y	Y	Y	Y	Y	5	High
14	El-Sherbini et al. [34]	Egypt	–	508	212	Microscopy	*Musca domestica*	*Hookworm* *T. trichura* *A. lumbricoides* *H. nana*	Y	N	Y	N	Y	3	Medium
15	Fetene et al. [35]	Ethiopia	December 2007–May 2008	430	111	Microscopy	*Musca domestica*	*C. parvum*	Y	Y	Y	Y	Y	5	High
16	Conn et al. [11]	America	June 2004–July 2004	4544	2886	Immunofluorescence technology	*Calliphora vicina, *	*Cryptosporidium *spp. *G. lamblia*	Y	Y	Y	Y	Y	5	High
17	Graczyk et al. [10]	America	–	250	25	Immunofluorescence technology	*Musca domestica*	*C. parvum*	Y	N	Y	N	Y	3	Medium
18	Graczyk et al. [36]	America	April 1999–September 1999	84	50	PCR	*Chrysomya megacephala*	*C. parvum*	Y	Y	Y	Y	N	4	High
19	Szostakowska et al. [37]	America	September 2003	104	28	Immunofluorescence technology	*Muscina stabulans,* *Lucilia cuprina* *Lucilia sericata* *Helicophagella melanura*	*C. parvum* *G. lamblia*	Y	Y	Y	Y	N	4	High
20	Lima et al. [38]	Brazil	October 2008–October.10	1180	472	Microscopy	*Musca domestica*	*E. histolytica* *E. coli* *Cystoisospora *spp. *Giardia *spp. *H. nana* *E. vermicularis* *T. trichiura* *Ascarids* *Taenia *spp. *I. butschlii*	Y	Y	Y	Y	Y	5	High
21	Oliveira et al. [39]	Brazil	May 1996–April 1998	2004	1144	Microscopy	*Musca domestica* *Chrysomya putoria* *Chrysomya albiceps* *Ophyra aenescens* *Fannia canicularis*	*A. lumbricoides* *T. leonina* *T. canis* *T. trichiura* *C. hepatica* *T. orientalis*	Y	Y	Y	Y	Y	5	High
22	Hemmati et al. [40]	Iran	November 2016–May 2017	210	126	PCR	*Musca domestica*	*E. granulosus*	Y	Y	Y	Y	Y	5	High
23	Yu et al. [41]	China	–	1000	120	PCR	*Musca domestica*	*E. bieneusi*	Y	N	Y	N	Y	3	Medium
24	Pornruseetriratn et al. [42]	Thailand	February 2013	60	48	PCR	*Musca domestica*	*T. solium* *T. saginata* *T. asiatica*	Y	Y	Y	Y	N	4	High
25	Zhao et al. [43]	China	July 2010–September 2010	800	160	PCR	*Musca domestica*	*C. parvum* *G. lamblia*	Y	Y	Y	Y	Y	5	High
26	Sulaiman et al. [44]	Malaysia	April 1985–September 1986	1418	628	Microscopy	*Musca domestica*	*A. lumbricoides* *T. trichiura* *Hookworm*	Y	Y	Y	Y	Y	5	High
27	Monzon et al. [45]	Philippines	August 1986–October 1986	1016	314	Microscopy	*Musca domestica, * *Chrysomya megacephala, *	*T. trichiura* *C. hepatica* *Hookworm* *A. lumbricoides* *T. canis* *T. solium*	Y	Y	Y	Y	Y	5	High
28	Barnes et al. [46]	Mongolia	April 2017–October 2017	115	17	PCR	*Lucilia cuprina, Chrysomya megacephala*	*G. duodenalis* *Cryptosporidium *spp.	Y	Y	Y	Y	N	4	High

*N* No,* Y* yes

^a^See footnote to Table 2 for full identification

### Statistical analysis

All statistical analyses were performed using Stata version 14.0 (StataCorp, College Station, TX, USA). Because there was heterogeneity in the data, heterogeneity of the study was determined as low heterogeneity (*I*^2^ < 25%), moderate heterogeneity (*I*^2^ = 25–75%) and high heterogeneity (*I*^2^ > 75%), and *P-*value < 0.05 was considered to be statistically significant [20]. Random effects models were used for the meta-analysis, including the sensitivity analysis, subgroup analysis and univariate regression analysis, to identify factors affecting heterogeneity. To evaluate the reliability of the data, we performed sensitivity analyses by removing individual studies one by one and combining other studies to assess the effect of selected studies on the pooled prevalence [47]. Forest plots were used to estimate differences across groups, and funnel plots and Egger’s tests were used to indicate possible publication bias in the study [48]. Potential sources of heterogeneity were assessed, including region (5 comparisons of continents), non-biting fly species (*Musca domestica* compared to other fly species), identification methods (morphology, molecular biology and immunofluorescence techniques), risk assessment (zoonotic and non-zoonotic parasites), non-biting fly body surface and gut (body surface only, gut and both combined prevalence), seasonality (four-season comparison) and parasite classification (protozoa compared to helminth) (Table 2).

**Table 2:** Analysis of the different groups based on the role of non-biting flies in the transmission of parasites

Potential sources of heterogeneity	No. of datasets	Sample size (*n*)	No. of samples that were positive	Prevalence, % (95% CI)	Heterogeneity			Univariate meta-regression		Correlation analysis Adjusted *R*^2^ (%)
					*χ*^2^	*P*-value	*I*^2^ (%)	*P*-value	Coefficient (95% CI)	
*Region*								0.800	− 0.101 (− 0.915 to 0.712)	− 3.58
Europe	7	2567	726	29.5 (13.2− 5.8)	1048.31	< 0.001	99.4			
Africa	8	13,366	9144	58.3 (47.4−69.3)	838.49	< 0.001	99.2			
North America	4	4982	2989	39.9 (6.4−73.5)	743.16	< 0.001	99.6			
Asia	7	3184	1616	36.9 (24.0−49.9)	629.30	< 0.001	99.0			
South America	2	4619	1413	48.6 (31.8−65.3)	89.80	< 0.001	98.9			
*Fly species*								0.624	0.192 (− 0.599 to 0.984)	− 2.42
Housefly (*Musca domestica*)	23	15,282	7234	43.3 (32.1−54.4)	5981.47	< 0.001	99.6			
Others^a^	10	13,436	8654	44.1 (23.9−64.3)	6199.00	< 0.001	99.9			
*Identification method*								0.424	0.282 (− 0.431 to 0.996)	− 1.27
Morphological identification	17	20,351	12,188	47.4 (33.0−61.7)	8338.75	< 0.001	99.8			
Molecular identification	8	3469	761	34.8 (24.6−45.1)	394.23	< 0.001	98.2			
Immunofluorescence technology	3	4898	2939	33.5 (7.1−74.1)	742.78	< 0.001	99.7			
*Risk assessment*								< 0.05	1.059 (0.428− 1.689)	21.78
Zoonotic parasite species	27	28,494	13,572	38.1 (28.2−48.0)	10,113.68	< 0.001	99.7			
Non-zoonosis	12	17,626	2316	13.3 (9.4−17.3)	638.36	< 0.001	98.3			
*Seasonality*								0.522	− 0.277 (-1.176 to 0.621)	− 3.49
Spring	1	9550	6685	70.0 (69.1−70.9)	NA	NA	NA			
Summer	9	9809	4331	29.7 (13.7−45.6)	2404.02	< 0.001	99.7			
Autumn	5	3003	1253	44.6 (31.7−57.5)	198.30	< 0.001	98.0			
Winter	3	522	121	42.0 (4.9−88.9)	322.66	< 0.001	99.4			
*Body surfaces and guts*								0.154	− 0.519 (− 1.245 to 0.207)	4.14
External surfaces	9	3649	1199	35.1 (20.8− 9.4)	1170.80	< 0.001	99.3			
Internal organs	7	3817	1045	37.1 (22.7−51.5)	741.44	< 0.001	99.2			
External surfaces/internal organs	12	21,252	13,644	51.1 (41.5−60.7)	2160.21	< 0.001	99.5			
*Parasite classification*								0.346	− 0.246 (− 0.770 to 0.278)	− 0.26
Protozoa	16	21,789	8097	32.1 (22.9−41.3)	3045.44	< 0.001	99.5			
Helminths	17	20,084	7791	42.6 (33.5−51.8)	3063.62	< 0.001	99.5			
Total	28	28,718	15,888	42.5 (31.9–53.2)	11,769.12	< 0.001	99.8			

*CI* Confidence interval,* NA* unknown/not available information

^a^*Cryptosporidium parvum*, Hookworm, *Strongyloides stercoralis*, *Ascaris lumbricoides*, *Taenia saginata*, *Trichuris trichiura*, *Taenia asiatica*, *Taenia solium*, *Ascaris suum*, *Metastrongylus apri*, *Strongyloides ransomi*, *Trichuris suis*, *Entamoeba histolytica*, *Giardia lamblia/duodenalis*, *Enterocytozoon bieneusi*, *Hymenolepis nana*, *Tricostrongilídeos orientalis*, *Echinococcus granulosus*, *Toxascaris leonine*, *Toxocara canis*, *Capillaria hepatica*, *Enterobius vermicularis*, *Trichuris vulpis*, *Ancylostoma caninum*, *Entamoeba coli*, *Iodamoeba butschlii, Cystoisospora spp.*

## Results

### Literature selection and research data extraction

Using the search strategy described above, 2193 studies were initially retrieved from the five databases (PubMed, 117 studies; PubMed Central, 841 studies; GeenMedical, 109 studies; Web of Science, 1113 studies; Science Direct, 13 studies). A total of 632 studies met the first round of screening criteria after deletion of duplicate articles in the databases. A total of 120 studies passed the second round of screening, with 512 studies whose titles and abstracts did not meet the selection criteria being excluded. Finally, 28 studies were identified for inclusion in the meta-analysis following review of the full text, with 92 studies excluded due to incomplete sample information (*n* = 20), incomplete data (*n* = 45) and review articles (*n* = 27) (Fig. 1).

**Fig. 2:**
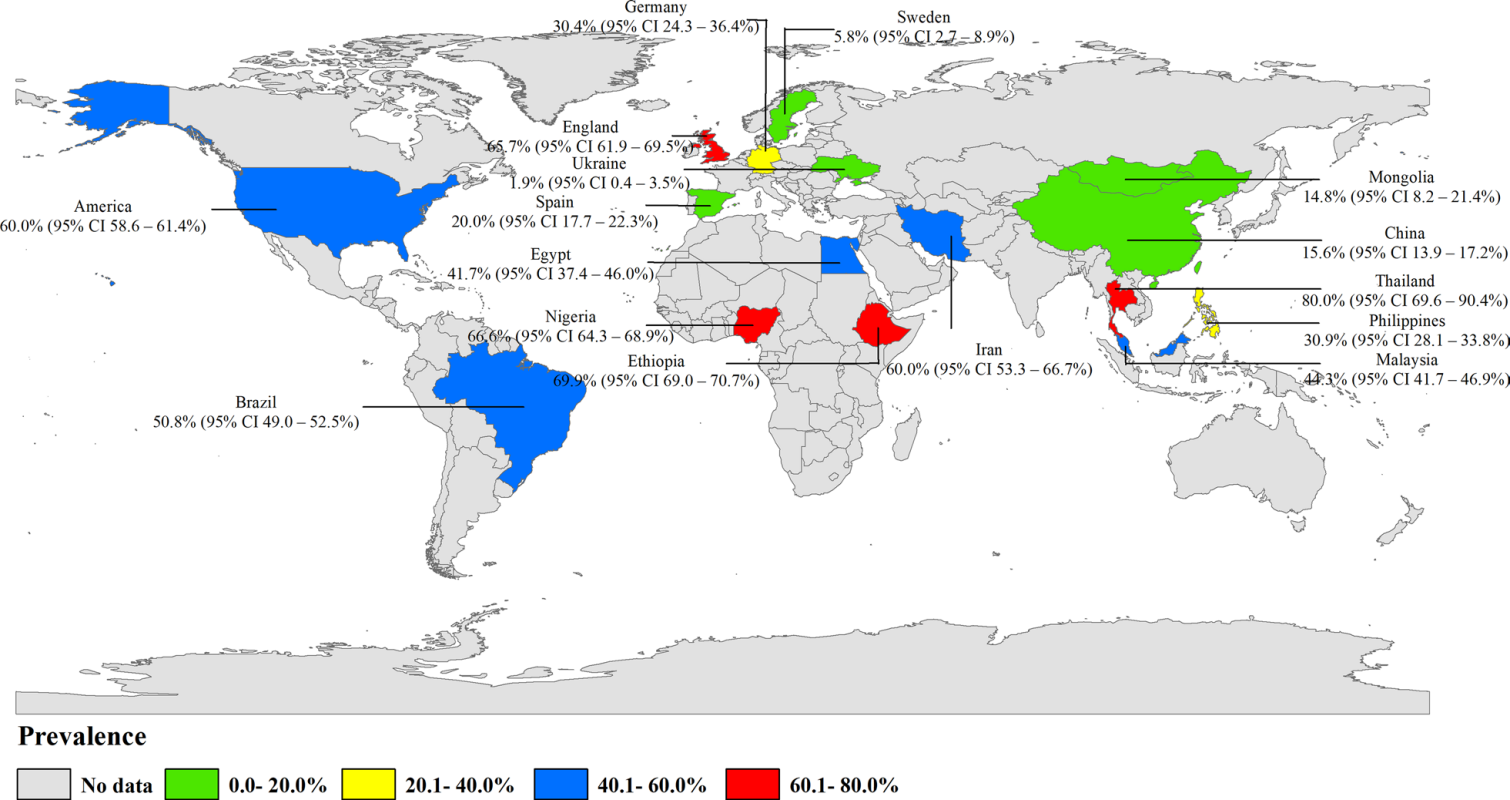
Prevalence and geographical distribution of non-biting flies carrying parasites. (This figure was originally designed using ArcGIS 10.4 software. The original vector diagram, imported in ArcGIS, was then adapted from Natural Earth (http://www.naturalearthdata.com). CI, Confidence interval

To date, 28 studies on the prevalence of parasites carried by non-biting flies cover 16 countries on five continents.(Fig. 2; Table 1). Among these, the highest number of studies were carried out in Africa, including Nigeria (*n* = 4), Ethiopia (*n* = 3) and Egypt (*n* = 1), followed by Asian countries, with seven studies, including China (*n* = 2), Philippines (*n* = 1), Iran (*n* = 1), Mongolia (*n* = 1), Malaysia (*n* = 1) and Thailand (*n* = 1). Seven studies were carried out in European countries, including Spain (*n* = 2), England (*n* = 2), Ukraine (*n* = 1), Germany (*n* = 1) and Sweden (*n* = 1). Those studies carried out in South America are mainly concentrated in Brazil (*n* = 2), and those carried out in North America are concentrated in the USA (*n* = 4) (Table 1). The prevalence and geographical distribution of parasites carried by non-biting flies are shown in Fig. 2. .

### Quality assessment

Evaluation of article quality showed that 21 of the 28 studies scored 4–5, indicating high quality, and seven studies scored 2–3, indicating moderate quality due to unclear sampling time and insufficient data on group analysis (Table 1).

### Sensitivity analysis and publication bias

**Fig. 3:**
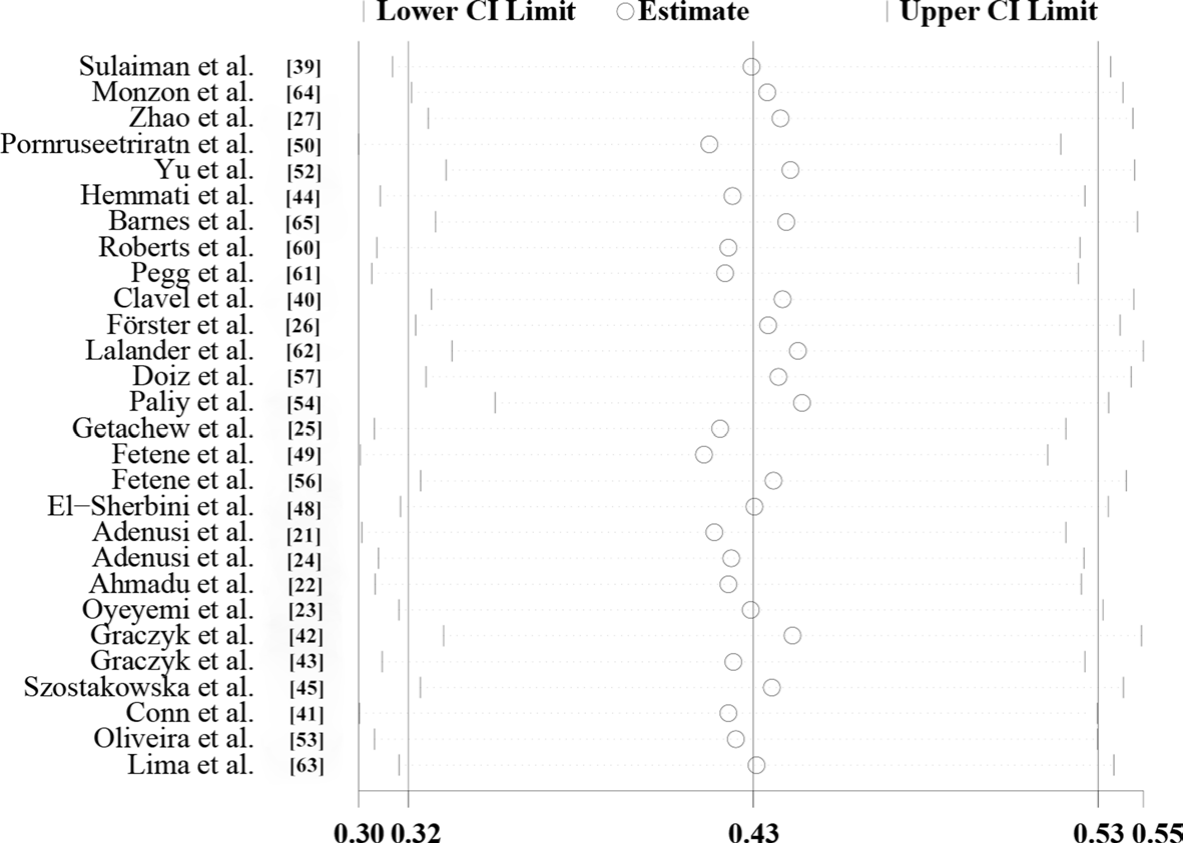
Sensitivity analysis of global prevalence of non-biting flies that have been found to transmit parasites

**Fig. 4:**
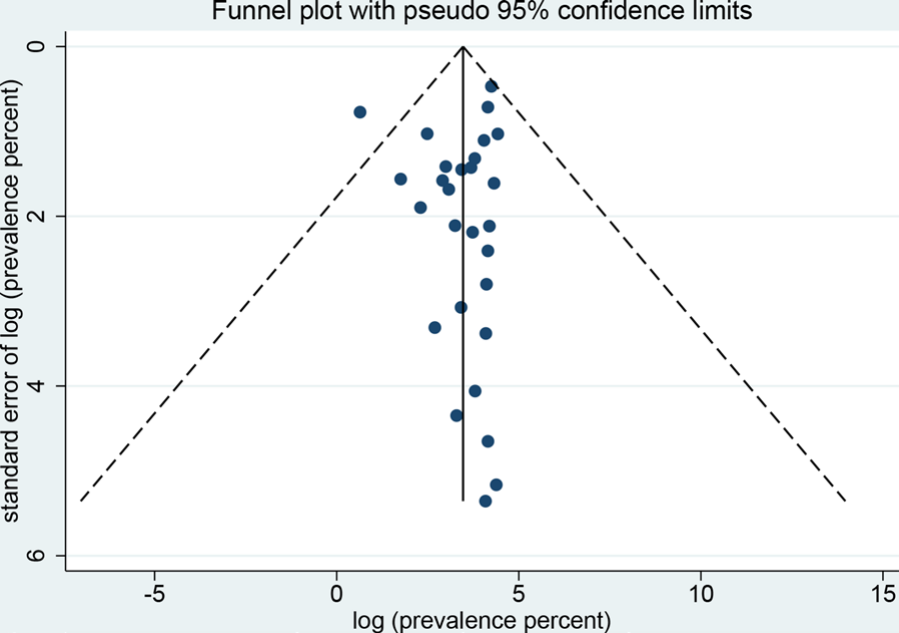
Funnel plot for the determination of publication bias of the global prevalence estimates of non-biting flies found to transmit parasites

The sensitivity analysis showed that the data were stable and the analysis was reliable (Fig. 3). Funnel plots were constructed to observe whether there was publication bias in the meta-analysis results. The plots showed that the effect points presented a basically symmetrical pattern and showed no publication bias (Fig. 4). Egger’s test (Table 3) was used to give *P* > 0.05, indicating that there was no publication bias in the data.

**Table 3:** Egger’s test for publication bias

StdEff^a^	Coefficient	Standard error	*t*	*P *> |*t*|	95% CI
Slope	57.587	9.193	6.26	0.000	38.690–76.484
Bias	− 9.206	7.000	− 1.32	0.200	− 23.595 to 5.182

^a^Standardized effects

### Different geographical distribution of parasites carried by non-biting flies

**Fig. 5:**
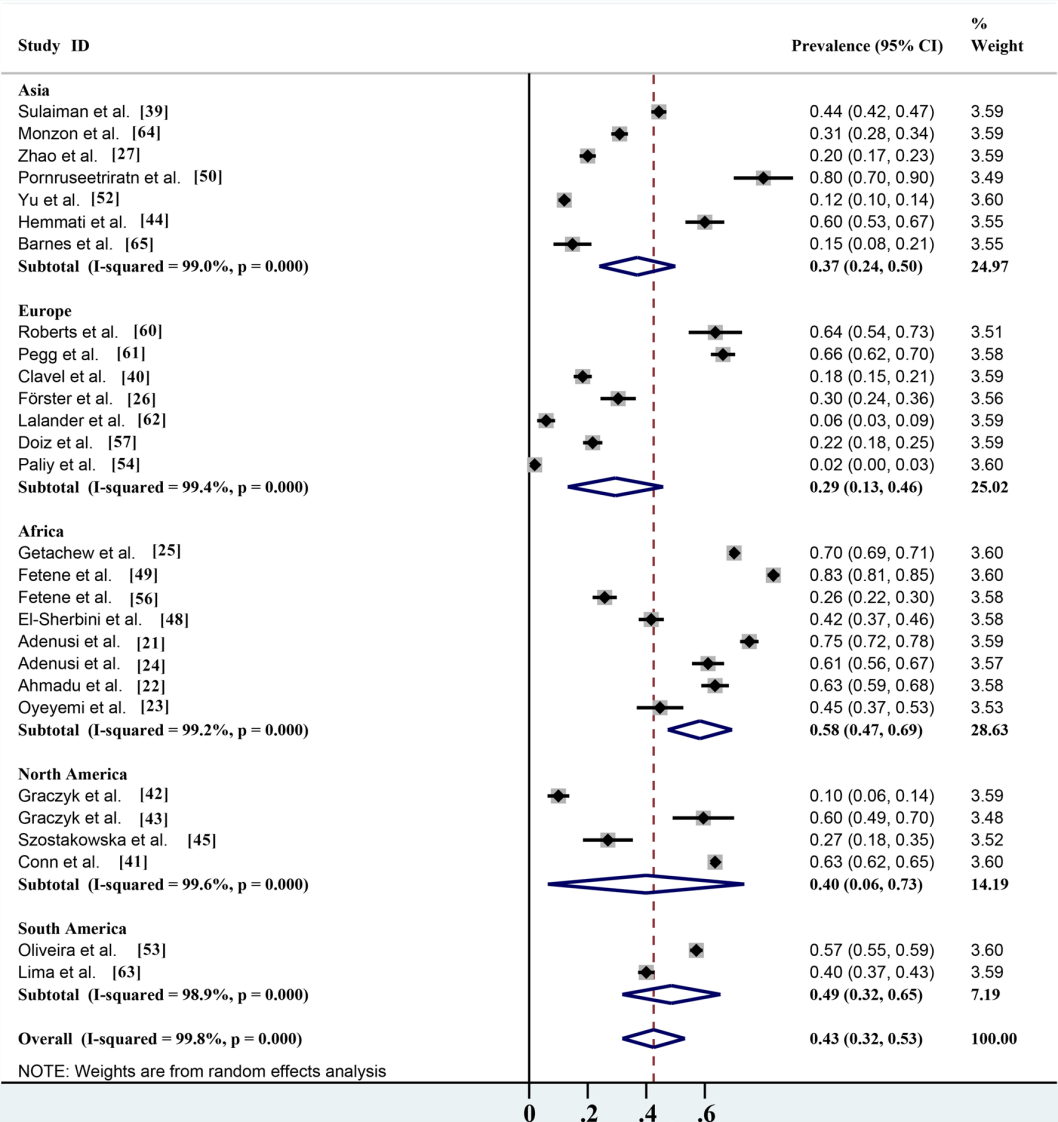
Forest plot of the global prevalence estimates of non-biting flies found to transmit parasites

The overall infection rate of parasites carried by non-biting flies worldwide is about 42.5% (95% confidence interval [CI] 31.9–53.2%;;* n* = 15,888/28,718) with heterogeneity (*I*^2^ = 99.8%, *P* < 0.001) (Table 2). Of the five continents reported in the literature, the highest number of studies were carried out in Africa, which also had the highest infection rate (58.3%; 95% CI 47.4–69.3%;;* n* = 9144/13,366) with heterogeneity (*I*^2^ = 99.2%, *P* < 0.001), followed by South America (48.6%; 95% CI 31.8–65.3%;* n* = 1413/4619) with heterogeneity (*I*^2^ = 98.9%, *P* < 0.001), North America (39.9%; 95% CI 6.4–73.5%;;* n* = 2989/4982) with heterogeneity (*I*^2^ = 99.6%, *P* < 0.001), Asia (36.9%; 95% CI 24.0–49.9%;;* n* = 1616/3184) with heterogeneity (*I*^2^ = 99.0%, *P* < 0.001) and Europe (29.5%; 95% CI 13.2–45.8%;;* n* = 726/2567) with heterogeneity (*I*^2^ = 99.4%, *P* < 0.001) (Table 2; Fig. 5). Among the 16 countries reported, Thailand had the highest infection rate (80.0%; 95% CI 69.6–90.4%;* n* = 48/60), and Ukraine has the lowest infection rate (1.9%; 95% CI 0.4–3.5%;* n* = 6/312) (Fig. 2).

### Pooled prevalence based on parasites carried by different non-biting flies

**Fig. 6:**
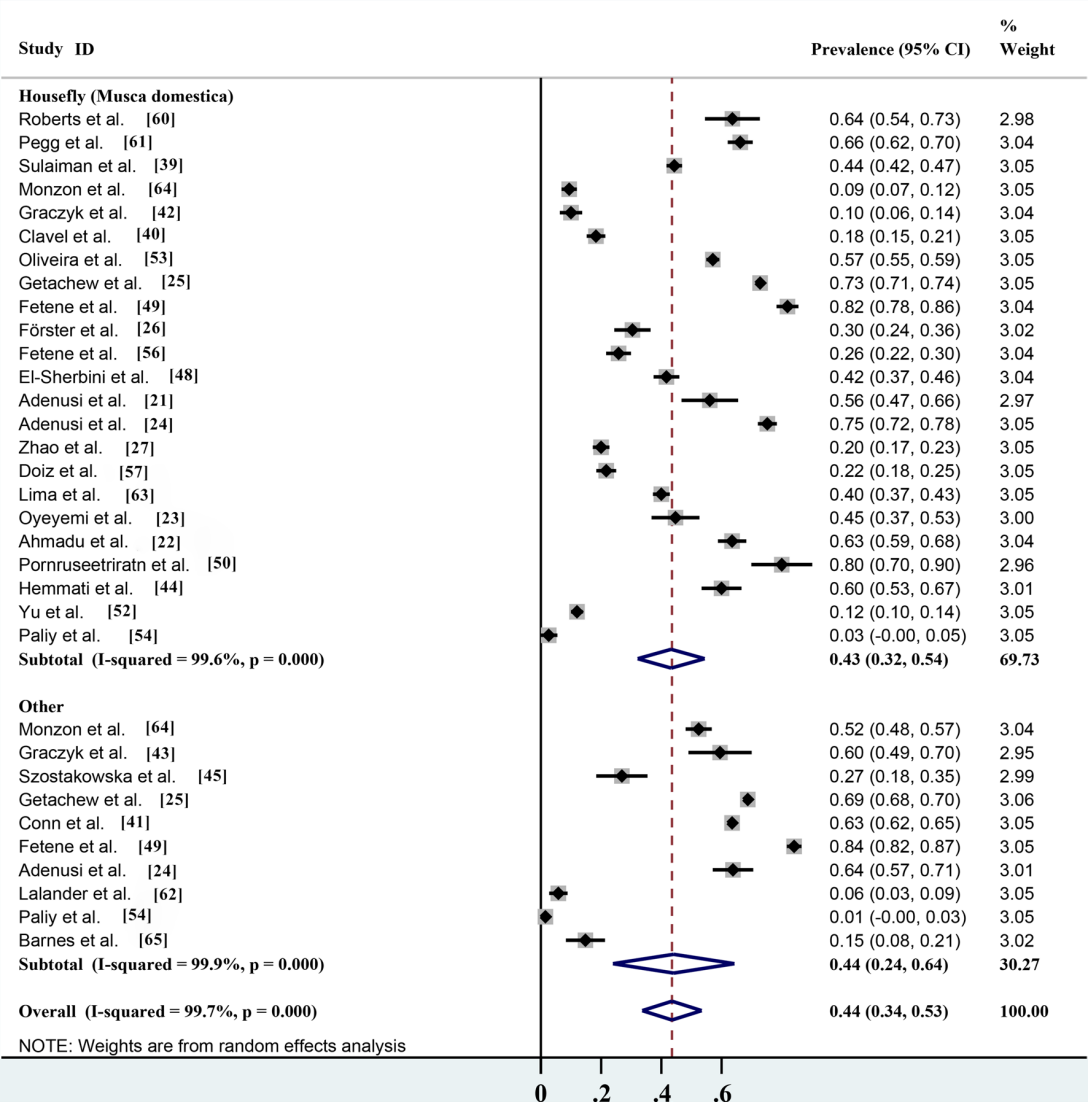
Forest plot of the prevalence estimates of parasites carried by different non-biting flies

Among the 28 studies, 23 reported that the house fly (*M. domestica*) carried parasites, which accounted for > 90% of all reported parasite species (Table 2). According to the statistics, the infection rate of parasites carried by the house fly (*M. domestica*) was 43.3% (95% CI 32.1–54.4%;* n* = 7234/15,282) with heterogeneity (*I*^2^ = 99.6%, *P* < 0.001), while the infection rate of parasites carried by the other 16 non-biting flies was 44.1% (95% CI 23.9–64.3%;* n* = 8654/13,436) with heterogeneity (*I*^2^ = 99.9%, *P* < 0.001) (Table 2; Fig. 6). These results showed that the house fly (*M. domestica*) was the most common fly species and its potential risk to human health could not be ignored. However, although other non-biting fly species are uncommon, their safety risks also cannot be ignored.

### Pooled prevalence based on different identification methods, risk assessment and seasonality

**Fig. 7:**
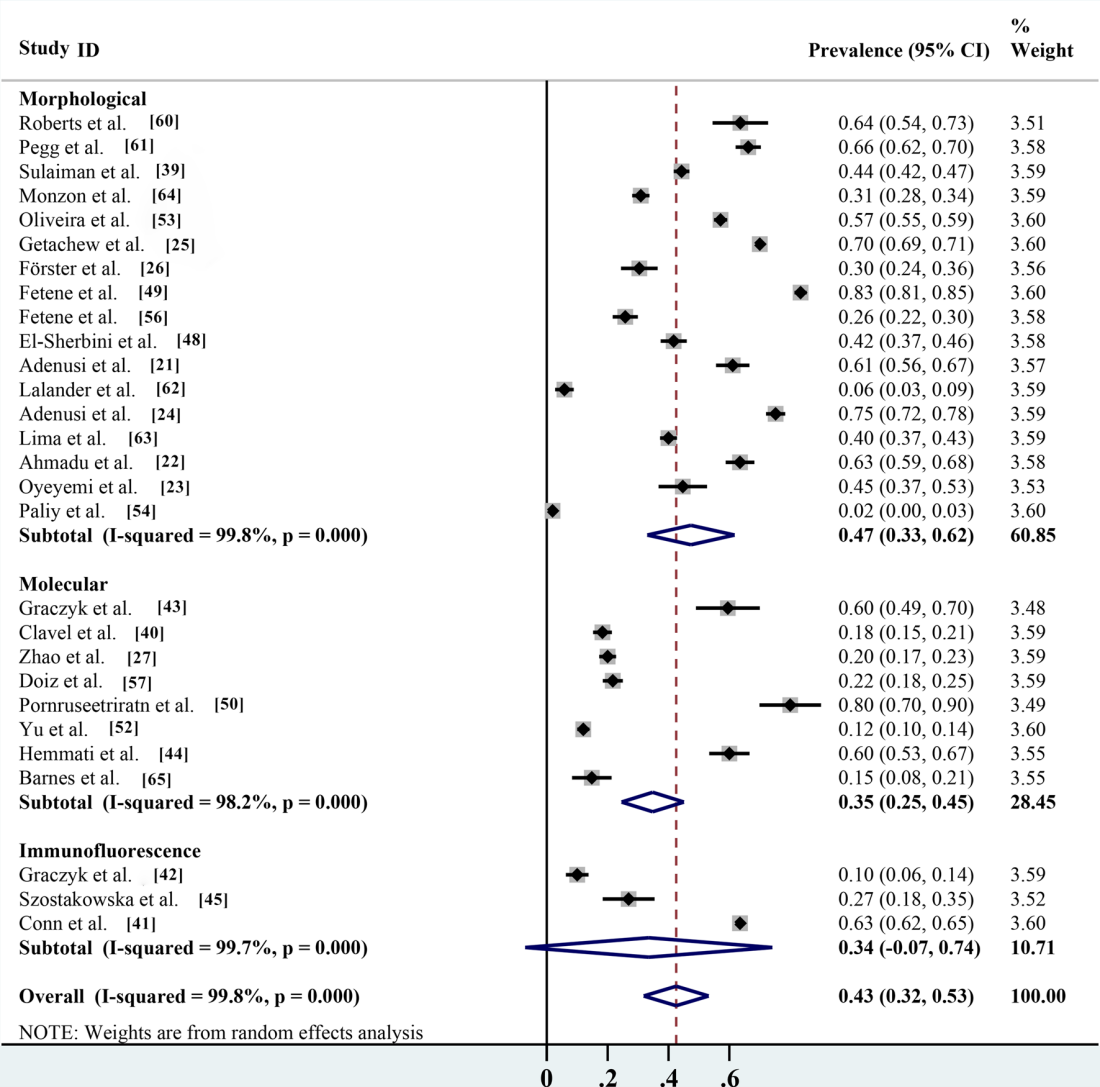
Forest plot of prevalence estimates for the different identification methods

**Fig. 8:**
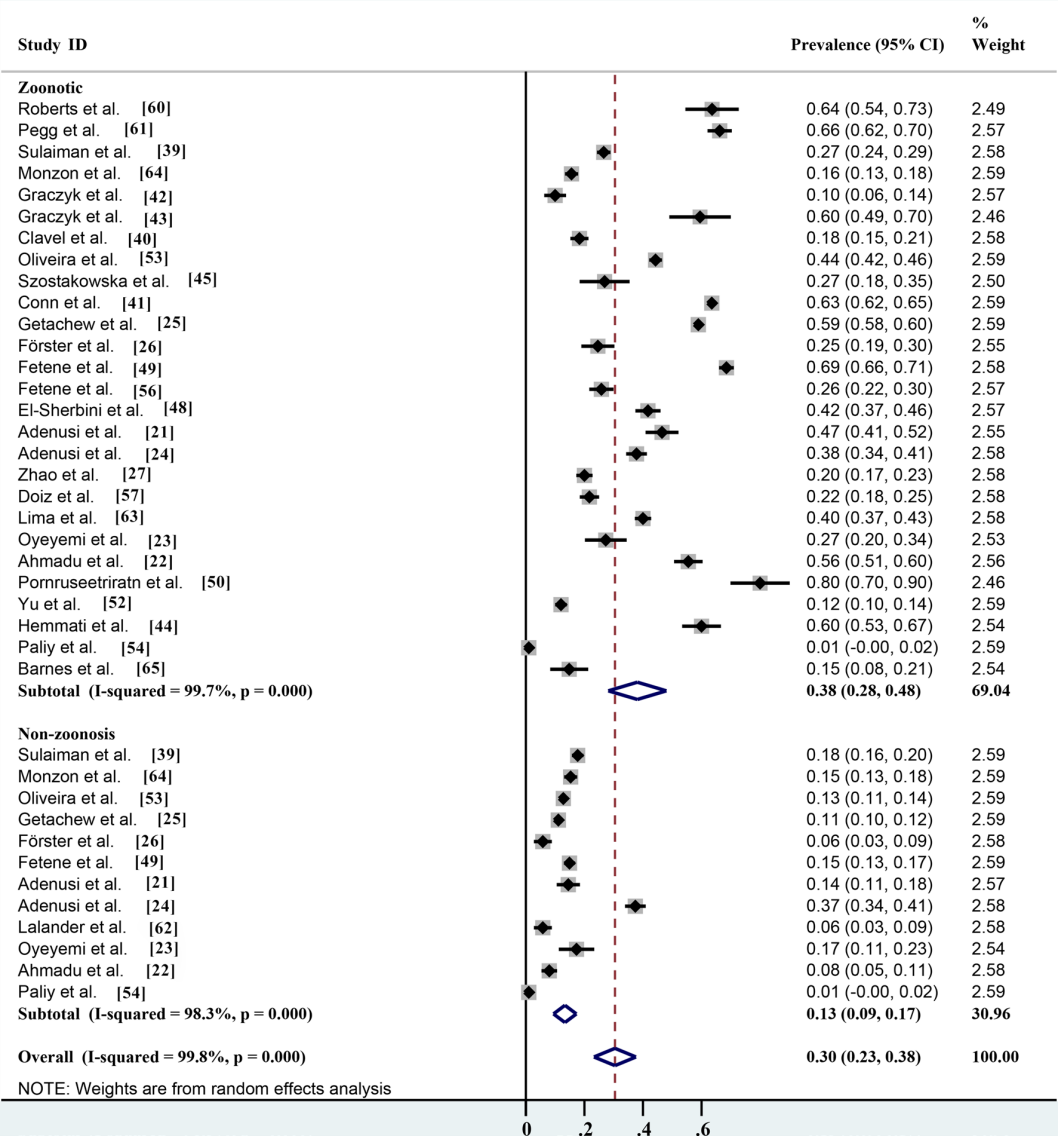
Forest plot of prevalence estimates for risk assessment of parasite transmission by non-biting flies

**Fig. 9:**
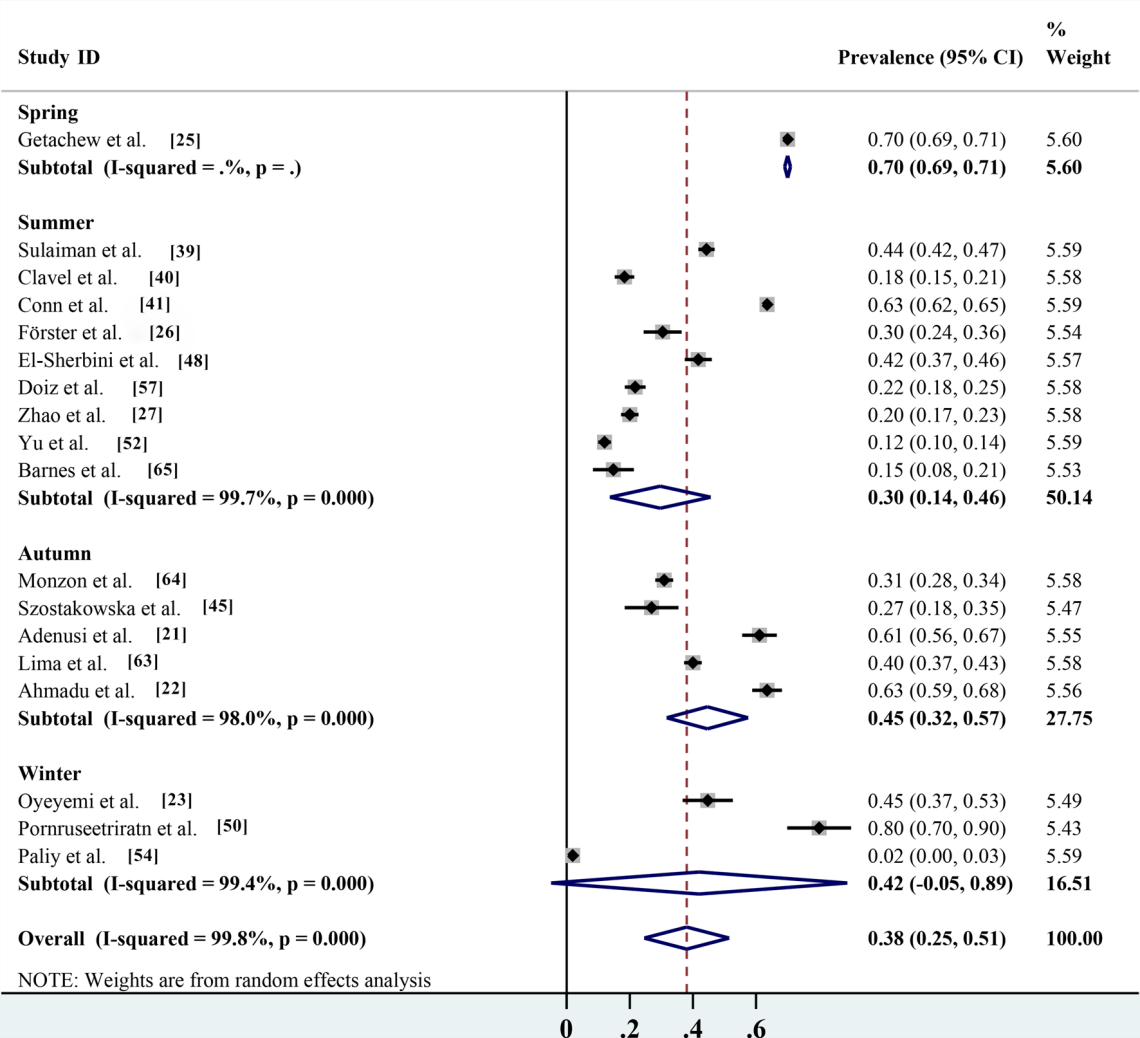
Forest plot of seasonal prevalence estimates of non-biting flies found to transmit parasites

Of the different identification methods listed in the studies, morphological identification is the most commonly used method for parasite identification; 17 of the 28 studies identified parasite species by microscopy, with an infection rate of 47.4% (95% CI 33.0–61.7%;* n* = 12,188/20,351) with heterogeneity (*I*^2^ = 99.8%, *P* < 0.001). The prevalence rates according to routine PCR molecular identification and immunofluorescence identification methods were 34.8% (95% CI 24.6–45.1%;* n* = 761/3469) with heterogeneity (*I*^2^ = 98.2%, *P* < 0.001) and 33.5% (95% CI 7.1–74.1%;* n* = 2939/4898) with heterogeneity (*I* ^2^ = 99.7%, *P* < 0.001), respectively (Table 2; Fig. 7). Non-biting flies are mechanical carriers of many parasitic species, most of which are zoonotic parasites. Among the 27 parasites reported, a total of 20 known zoonotic parasites were identified. The infection rate was 38.1% (95% CI 28.2–48.0%;* n* = 13,572/28,494) with heterogeneity (*I*^2^ = 99.7%, *P* < 0.001), and the prevalence rate of non-zoonotic parasitic diseases was 13.3% (95% CI 9.4–17.3%;* n* = 2316/17,626) with heterogeneity (*I*^2^ = 98.3%, *P* < 0.001) (Table 2; Fig. 8). The species of parasites carried by non-biting flies can differ seasonally. The highest infection rate was 70.0% (95% CI 69.1–70.9%;* n* = 6685/9550) and occurred in the spring, followed by the autumn, winter and summer, with infection rates of 44.6% (95% CI 31.7–57.5%;* n* = 1253/3003) with heterogeneity (*I*^2^ = 98.0%, *P* < 0.001), 42.0% (95% CI 4.9–88.9%;* n* = 121/522) with heterogeneity (*I*^2^ = 99.4%, *P* < 0.001) and 29.7% (95% CI 13.7–45.6%;* n* = 4331/9809) with heterogeneity (*I* ^2^ = 99.7%, *P* < 0.001), respectively (Table 2; Fig. 9).

### Pooled prevalence based on parasites carried on the body surface and guts of non-biting flies

**Fig.10:**
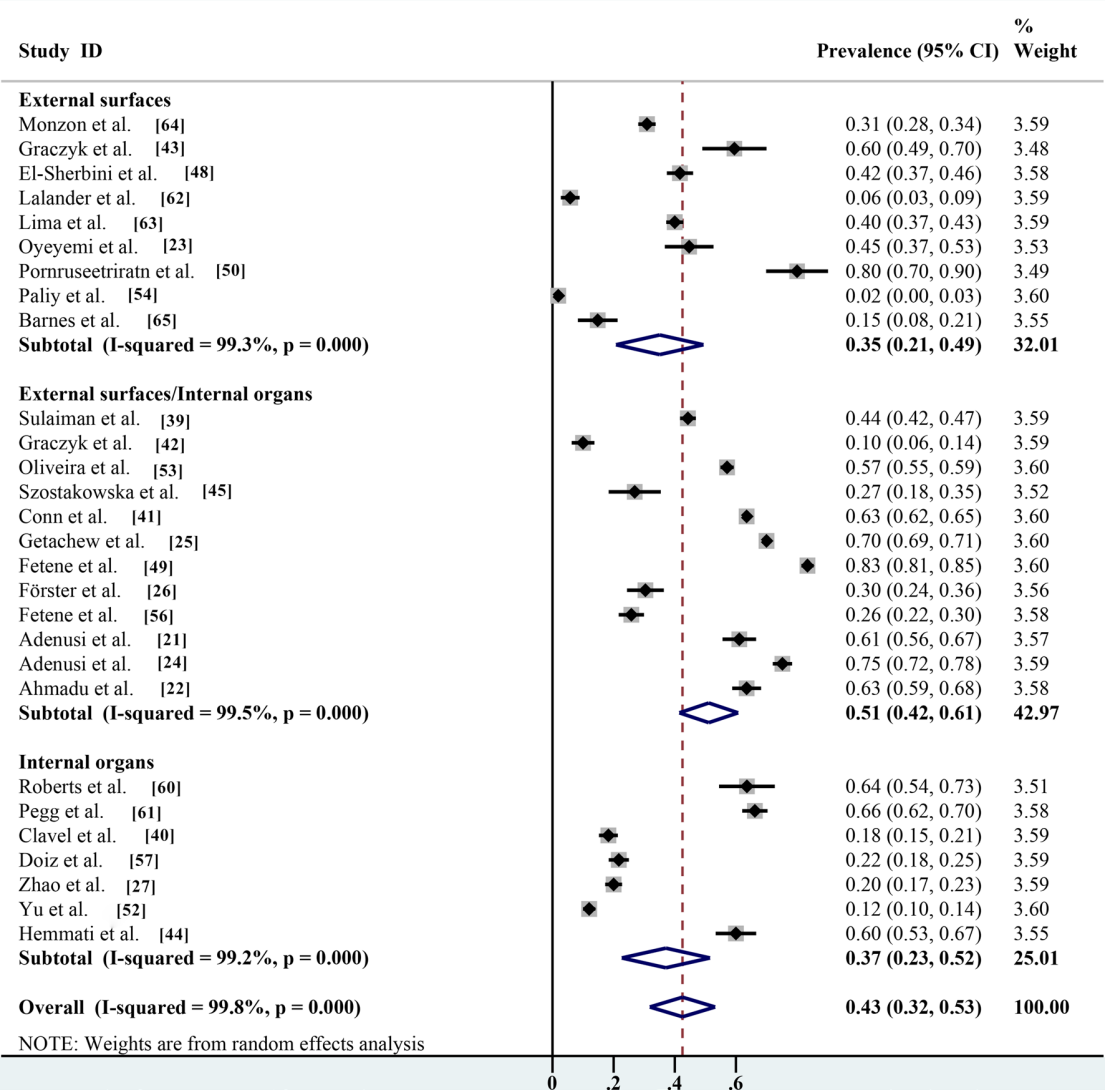
Forest plot for estimation of prevalence of parasites carried on the body surface and gut of non-biting flies

Non-biting flies can infect humans and animals by contacting and adhering to parasite eggs/cysts through mouthparts and body hair covering their entire body. They can also indirectly infect humans and animals through intestinal excretion of contaminated water and body waste. Several studies have shown that parasites can be isolated from both the body surface and gut of non-biting flies, with a prevalence of 51.1% (95% CI 41.5–60.7%;* n* = 13,644/21,252) with heterogeneity (*I*^2^ = 99.5%, *P* < 0.001). The prevalence of parasites isolated from the intestine of the non-biting flies investigated only was 37.1% (95% CI 22.7–51.5%; 1045/3817) with heterogeneity (*I*^2^ = 99.2%, *P* < 0.001), which was significantly higher than the prevalence of parasites isolated from the body surface (35.1%; 95% CI 20.8–49.4%;* n* = 1199/3649) with heterogeneity (*I*^2^ = 99.3%, *P* < 0.001) (Table 2; Fig. 10).

### Pooled prevalence based on protozoa cysts and helminths eggs carried by non-biting flies

**Fig. 11:**
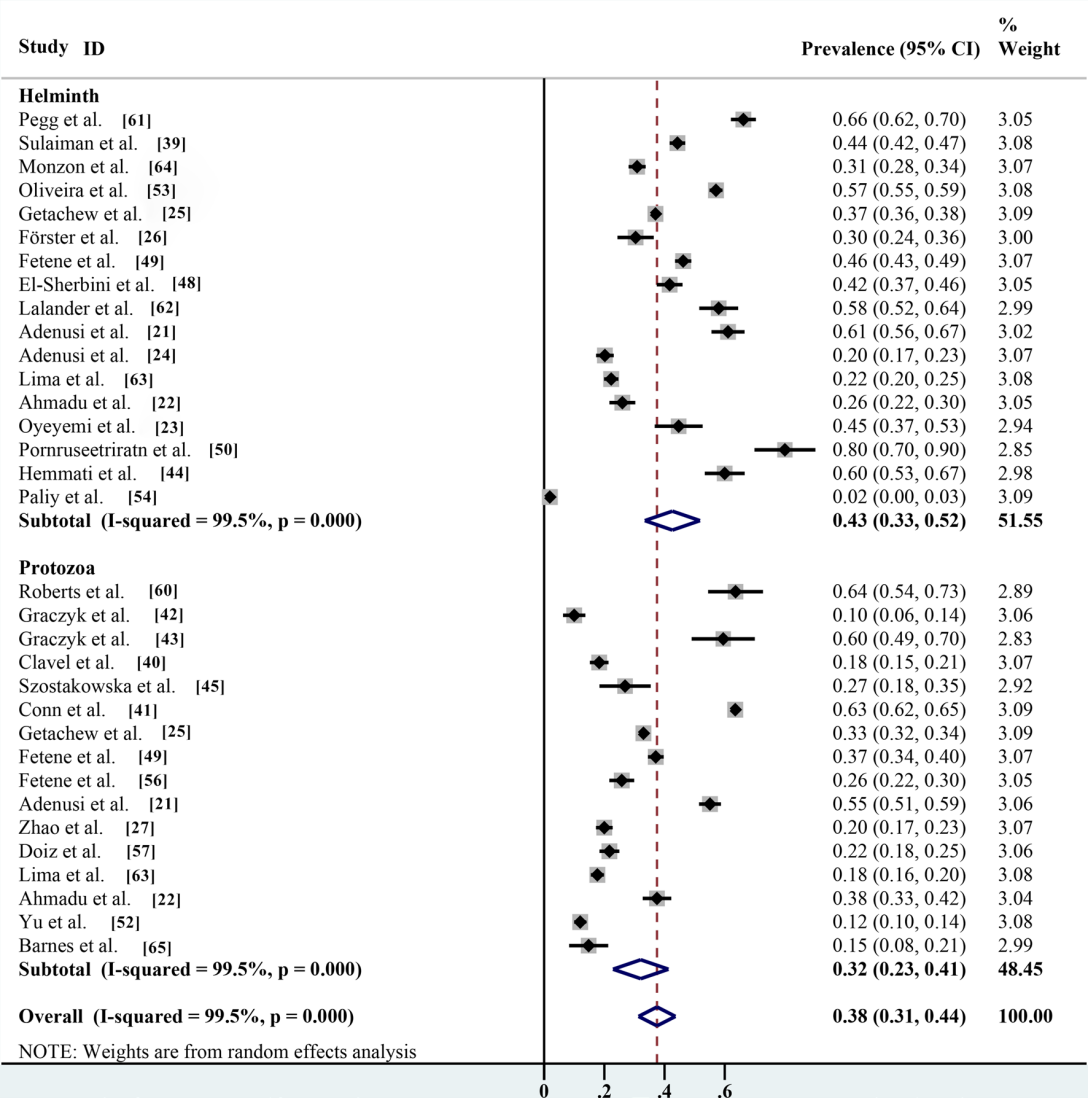
Forest plot of global estimated prevalence of protozoa and helminths carried by non-biting flies

According to the subgroup analysis (protozoa compared with helminths), the prevalence of helminth eggs carried by non-biting flies was 42.6% (95% CI 33.5–51.8%;* n* = 7791/20,084) with heterogeneity (*I*^2^ = 99.5%, *P* < 0.001) was significantly higher than that of protozoa cysts (32.1%; 95% CI 22.9–41.3%;* n* = 8097/21,789) with heterogeneity (*I*^2^ = 99.5%, *P* < 0.001) (Table 2; Fig. 11).

### Sources of heterogeneity by meta-regression analysis

All studies included in this meta-analysis had significant heterogeneity, and the source of heterogeneity was further determined by univariate regression analysis. The results showed whether or not zoonotic parasites (*P* < 0.05) were the key factors of heterogeneity (Table 2).

## Discussion

Non-biting flies are common on farms and in residential areas and have a close relationship with humans and animals [41]. Feces, garbage and sewage attract non-biting flies and are often the most suitable locations for reproduction [33, 43]. Non-biting flies mainly pollute water sources, fruits and vegetables and animal feed through body surface contact and intestinal excretions [28, 32]. Humans and animals are indirectly infected by eating food and water containing parasitic eggs or cysts. In addition to transmission through water and food, non-biting flies can also spread parasitic eggs/cysts through contact with human and animal skin, which increases the risk of human and animal infection [33, 31].

This systematic review included 28 studies covering 16 countries across five continents. The reliability of estimated prevalence of parasites carried by non-biting flies worldwide was derived by meta-analysis. The highest prevalence was found in Afric (58.3%; 95% CI 47.4–69.3%;* n* = 9144/13,366); two studies, one each in South America and North America were reported, but these were not representative [38]. Prevalence is reported to be generally high in African and Asian countries, such as Ethiopia (69.9%; 95% CI 69.0–70.7%;* n* = 7885/11,286) [29], Nigeria (66.6%;(95% CI 64.3–68.9%;* n* = 1047/1572) [30], Iran (60.0%; 95% CI 53.3–66.7%;* n* = 126/210) [40] and Thailand (80.0%; 95% CI 69.6–90.4%;* n* = 48/60) [42]. In contrast, the prevalence ranged from 1.9% to 65.7% in European countries [24, 25, 27], from 41.7% to 69.9% in African countries and from 14.8% to 80.0% in Asian countries, mainly due to environmental factors affecting health [26]. According to the literature, most feces and garbage in Africa are handled improperly, and open-air defecation is prevalent in children and a small number of adults [29].

Different non-biting fly species are likely to carry various kinds of parasites [49]. Individual non-biting flies can carry ≥ 2 parasites, and eggs of the same parasites, such as hookworm, *Ascaris lumbricoides* and *Trichuris trichiura* can be isolated from different species of flies [39]. The house fly *(M. domestica)* are common in areas of human habitation and are closely associated with human activities [17]; as such, they are most likely involved in parasite transmission. In general, studies have focused on the house fly (*M. domestica*), including some laboratory studies and surveys of the prevalence of transmitted parasites. However, although other fly species are not as abundant in human habitations and animal environments as the house fly (*M. domestica*), the prevalence of parasites carried by these fly species has shown a linear increase in some countries with poor sanitary conditions, such as Africa and Asia [45].

Methods are available for the isolation and identification of parasites carried on the body surface and intestine of non-biting flies. The parasite species can be identified by morphological observation of eggs/cysts under magnification by optical microscopy. They can also be identified by molecular methods and immunofluorescence techniques [50]. Morphological identification is the most commonly used method for parasite identification, with prevalence statistics reaching 47.4% (95% CI 33.0–61.7%;* n* = 12,188/20,351) [51, 52]. However, most parasite eggs/cysts share similar morphological characteristics, and in most cases species cannot be identified using light microscopy [53]. In addition, microscopy may overestimate the prevalence due to the long publication time of multiple studies, mostly concentrated in Africa, in some countries with limited experimental conditions. To address this problem, molecular techniques are a viable alternative for identifying parasite species [54, 55]. Molecular identification is mainly based on conventional PCR, which has the advantages of strong specificity, high sensitivity, easy operation and low cost [56]. The identification of parasites using genetic characteristics is now widely applied, and species identified in this way include *Cryptosporidium* spp. and *Giardia lamblia* [35, 23, 46]. *Cryptosporidium* spp. can be accurately identified based on its small subunit ribosomal RNA (SSU rRNA) gene [57]. The 60-kDa glycoprotein gene (*gp60*) is the most commonly used gene locus in *Cryptosporidium* spp. genotyping [58]. Commonly used gene loci for genotyping *G. lamblia* are SSU rRNA [59], β-giardin (*bg*), glutamate dehydrogenase (*gdh*) and triose-phosphate isomerase (*tpi*) [60, 61, 44]. Immunofluorescence technology combines a fluorescent-labeled antibody or antigen with the corresponding antigen or antibody in the test sample and detects fluorescence under a microscope. It is a reliable, rapid, sensitive and widely applicable application and can detect *Cryptosporidium parvum* oocysts [21].

Non-biting flies are mechanical carriers of many parasitic species, most of which are zoonotic parasites [40, 37, 62]. In addition to farms, the environments used for sample collection included residential areas, restaurants and fruit and vegetable stores [1]. The sequences of the SSU rRNA gene and the gp60 locus of *C. parvum* in non-biting flies were 100% homologous with the sequences of *C. parvum* from humans, indicating that non-biting flies were likely vectors of *C. parvum* [63]. The IIdA19G1 subtype identified in non-biting flies was the same as that of found in cattle from the same dairy farm studied [43, 34]. Non-biting flies will therefore increase the risk of *Cryptosporidium* infection in humans. The present study provided evidence for assessing the role of non-biting flies as transport hosts of parasites in the transmission of parasitic diseases.

The species of parasites carried by non-biting flies can differ seasonally. The seasonal growth and decline of non-biting flies show a three-peak curve. The highest peak, sub-peak and minimum peak appear in late May, early March and early September, respectively [11]. Non-biting flies pass through egg, larva, pupa and adult stages, and the developmental rates of these stages depend on temperature [10, 36]. Under favorable conditions in the summer and autumn, the development from egg to adult fly can be completed in 7 days. This rapid generation time explains why the fly populations sometimes grow explosively under warm conditions [31]. An increase in the number of flies can increase parasite prevalence in humans and animals [32, 33]. Although the prevalence rate was found to be highest in the spring, only one study reported this result, and there is no reference value: despite a large sample size, the detection method was by microscopic observation, and mistakes are likely when this method is used to identify protozoal parasites [31]. The second-higest prevalence was found in the autumn, as reported by five studies; this conclusion was reliable according to the seasonal fluctuation law [30, 28]. Finally, although the infection rate was lowest in the summer, nine studies reported this result; however, this conclusion was not credible according to the law of seasonal waxing and waning, and most of these nine studies focused on European countries with better sanitary conditions compared with the studies in the spring and autumn studies which were carried out in African countries [30, 21, 11].

Several studies combined the prevalence of parasites carried on the body surface and gut of non-biting flies (51.1%; 95% CI 41.5–60.7%;* n* = 13,644/21,252), and combined prevalence was selected for the statistical analysis in the present meta-analysis because data could not be subdivided [22, 64]. In addition, it has been shown that the prevalence of parasites isolated from the gut of non-biting flies alone is 37.1% (95% CI 22.7–51.5%;* n* = 1045/3817), which is significantly higher than the prevalence of parasites isolated from the body surface (35.1%; 95% CI 20.8–49.4%;* n* = 1199/3649). These results indicate that parasites were more easily transmitted by intestinal excretion. The results of this study are consistent with previous findings in Nigeria on the potential risk of transmission of human intestinal helminths eggs by non-biting flies [30] and with previous studies in Ethiopia that investigated the transmission of intestinal helminths eggs by non-biting flies in residential areas [31].

The most common methods used to identify helminths eggs are mostly morphological ones, and egg morphology is observed using a microscope. Identification can be directly made because some eggs have specific characteristics, with a prevalence of 42.6% (95% CI 33.5–51.8%). However, for the detection of protozoa, morphological identification alone cannot be used to make an accurate judgment, and even fungal spores can be mistaken for protozoa, with a prevalence of 32.1% (95% CI 22.9–41.3%). In some publications, only *Cryptosporidium* spp. and *Giardia* spp. were observed by microscopy, and no specific species could be identified; therefore, the best way to distinguish parasite species is by molecular tools [31, 29]. Most studies identified *Entamoeba histolytica* and *Escherichia coli* by morphological observation and did not apply molecular tools to distinguish them; consequently, the prevalence of *E. histolytica* has likely been overestimated [30, 28].

Although this systematic review covers studies reporting on non-biting flies that transmit parasites in multiple countries, there are a number of limitations. First, some of the studies/publications identified during the search could not be downloaded and were therefore not included in the analysis [65]. Second, the publication of relevant articles spans many years, there are only a few such published studies and there is a lack of understanding of the prevalence of parasites carried by non-biting flies. Third, the identification methods for detecting parasites are limited, and published studies mostly use conventional microscopic identification, with the likelihood that some prevalence rates may be overestimated. However, even with these limitations, the purpose of using meta-analysis in this study was to increase the sample size and power of the meta-analysis, so that the study results were close to the true prevalence.

## Conclusion

Available studies have shown that the prevalence of parasites transmitted by non-biting flies worldwide is relatively high at 42.5%, and associated risk factors, such as zoonotic risk, should be considered so that people can implement effective management plans according to local conditions that may differ between geographical regions and environments, and prevent zoonotic transmission. Non-biting flies are mechanical vectors of a variety of parasites, most of which are zoonotic parasites, which can circulate between humans and humans, and between humans and animals as vectors. Thus, fly vectors should be controlled, especially in human residential areas and farms. This study provides a theoretical basis for the public health and ecological significance of parasites transmitted by non-biting flies. Future studies should mostly use molecular diagnostic tools because it not only improves detection rates, but also accurately distinguishes parasite species and reduces errors.

## Supplementary Information

**Additional file 1: Table S1.** PRISMA checklist.

## Data Availability

All data used and analyzed in this study are represented in Tables 1, 2, 3.

## Abbreviations

*bg*: β-Giardin gene
*gdh*: Glutamate dehydrogenase gene
PRISMA: Preferred Reporting Items for Systematic Reviews and Meta-Analyses
SSU rRNA: Small subunit ribosomal RNA
*tpi*: Triose-phosphate isomerase gene

## References

[1] Patel A, Jenkins M, Rhoden K, Barnes AN (2022). A systematic review of zoonotic enteric parasites carried by flies, cockroaches, and dung beetles. Pathogens.

[2] Ranjbar R, Izadi M, Hafshejani TT, Khamesipour F (2016). Molecular detection and antimicrobial resistance of Klebsiella pneumoniae from house flies (*Musca domestica*) in kitchens, farms, hospitals and slaughterhouses. J Infect Public Health.

[3] Park R, Dzialo MC, Spaepen S, Nsabimana D, Gielens K, Devriese H (2019). Microbial communities of the house fly *Musca domestica* vary with geographical location and habitat. Microbiome.

[4] Adenusi AA, Akinyemi MI, Akinsanya D (2018). Domiciliary cockroaches as carriers of human intestinal parasites in Lagos Metropolis, Southwest Nigeria: implications for public health. J Arthropod Borne Dis.

[5] Pava-Ripoll M, Pearson RE, Miller AK, Ziobro GC (2015). Detection of foodborne bacterial pathogens from individual filth flies. J Vis Exp.

[6] Förster M, Klimpel S, Mehlhorn H, Sievert K, Messler S, Pfeffer K (2007). Pilot study on synanthropic flies (e.g. Musca, Sarcophaga, Calliphora, Fannia, Lucilia, Stomoxys) as vectors of pathogenic microorganisms. Parasitol Res..

[7] Baldacchino F, Muenworn V, Desquesnes M, Desoli F, Charoenviriyaphap T, Duvallet G (2013). Transmission of pathogens by Stomoxys flies (Diptera, Muscidae): a review. Parasite.

[8] Mohd AK (2018). A review on respiratory allergy caused by insects. Bioinformation.

[9] Stoffolano JG (2019). Fly foregut and transmission of microbes. Adv In Insect Phys.

[10] Graczyk TK, Cranfield MR, Fayer R, Bixler H (1999). House flies (*Musca domestica*) as transport hosts of *Cryptosporidium parvum*. Am J Trop Med Hyg.

[11] Conn DB, Weaver J, Tamang L, Graczyk TK (2007). Synanthropic flies as vectors of *Cryptosporidium* and *Giardia* among livestock and wildlife in a multispecies agricultural complex. Vector Borne Zoonotic Dis.

[12] Khamesipour F, Lankarani KB, Honarvar B, Tebit KE (2018). A systematic review of human pathogens carried by the housefly (*Musca domestica* L.). BMC Public Health..

[13] Matthews K (2011). Controlling and coordinating development in vector-transmitted parasites. Science.

[14] Tatfeng YM, Usuanlele MU, Orukpe A, Digban AK, Okodua M, Oviasogie F (2005). Mechanical transmission of pathogenic organisms: the role of cockroaches. J Vector Borne Dis.

[15] Moher D, Liberati A, Tetzlaff J, Altman DG, PRISMA Group (2009). Preferred reporting items for systematic reviews and meta-analyses: the PRISMA statement. PLoS Med..

[16] Patramool S, Choumet V, Surasombatpattana P, Sabatier L, Thomas F, Thongrungkiat S (2012). Update on the proteomics of major arthropod vectors of human and animal pathogens. Proteomics.

[17] Barreiro C, Albano H, Silva J, Teixeira P (2013). Role of flies as vectors of foodborne pathogens in rural areas. ISRN Microbiol.

[18] Page MJ, McKenzie JE, Bossuyt PM, Boutron I, Hoffmann TC, Mulrow CD (2021). The PRISMA 2020 statement: an updated guideline for reporting systematic reviews. BMJ.

[19] Guyatt GH, Oxman AD, Vist GE, Kunz R, Falck-Ytter Y, Alonso-Coello P (2008). GRADE: an emerging consensus on rating quality of evidence and strength of recommendations. BMJ.

[20] Badri M, Olfatifar M, Wandra T, Budke CM, Mahmoudi R, Abdoli A (2022). The prevalence of human trichuriasis in Asia: a systematic review and meta-analysis. Parasitol Res.

[21] Clavel A, Doiz O, Morales S, Varea M, Gómez-Lus R (2002). House fly (*Musca domestica*) as a transport vector of *Cryptosporidium parvum*. Folia Parasitol.

[22] Förster M, Klimpel S, Sievert K (2009). The house fly (*Musca domestica*) as a potential vector of metazoan parasites caught in a pig-pen in Germany. Vet Parasitol.

[23] Doiz O, Clavel A, Morales S, Varea M, Castillo FJ, Rubio C (2000). House fly (*Musca domestica*) as a transport vector of *Giardia lamblia*. Folia Parasitol.

[24] Roberts EW (1947). The part played by the faeces and vomit-drop in the transmission of *Entamoeba histolytica* by *Musca domestica*. Ann Trop Med Parasitol.

[25] Pegg EJ (1971). Infection of dogs by *Toxocara canis* carried by flies. Parasitology.

[26] Paliy A, Sumakova N, Mashkey A, Petrov R, Ishchenko K (2018). Contamination of animal-keeping premises with eggs of parasitic worms. Biosyst Divers.

[27] Lalander C, Diener S, Magri ME, Zurbrügg C, Lindström A, Vinnerås B (2013). Faecal sludge management with the larvae of the black soldier fly (Hermetia illucens)—from a hygiene aspect. Sci Total Environ.

[28] Ahmadu YM, Goselle ON, Ejimadu LC, James Rugu NN (2016). Microhabitats and pathogens of houseflies (*Musca domestica*). J Biol..

[29] Fetene T, Worku N (2009). Public health importance of non-biting cyclorrhaphan flies. Trans R Soc Trop Med Hyg.

[30] Adenusi AA, Adewoga TO (2013). Human intestinal parasites in non-biting synanthropic flies in Ogun State. Nigeria Travel Med Infect Dis.

[31] Getachew S, Gebre-Michael T, Erko B, Balkew M, Medhin G (2007). Non-biting cyclorrhaphan flies (Diptera) as carriers of intestinal human parasites in slum areas of Addis Ababa, Ethiopia. Acta Trop.

[32] Oyeyemi OT, Agbaje MO, Okelue UB (2016). Food-borne human parasitic pathogens associated with household cockroaches and houseies in Nigeria. Parasite Epidemiol Control.

[33] Adenusi AA, Adewoga TO (2013). Studies on the potential and public health importance of non-biting synanthropic flies in the mechanical transmission of human enterohelminths. Trans R Soc Trop Med Hyg.

[34] El-Sherbini GT, Gneidy MR (2012). Cockroaches and flies in mechanical transmission of medical important parasites in Khaldyia Village, El-Fayoum, Governorate, Egypt. J Egypt Soc Parasitol.

[35] Fetene T, Worku N, Huruy K, Kebede N (2011). *Cryptosporidium* recovered from *Musca domestica*, *Musca sorbens* and mango juice accessed by synanthropic flies in Bahirdar, Ethiopia. Zoonoses Public Health.

[36] Graczyk TK, Fayer R, Knight R, Mhangami-Ruwende B, Trout JM, Da Silva AJ (2000). Mechanical transport and transmission of *Cryptosporidium parvum* oocysts by wild filth flies. Am J Trop Med Hyg.

[37] Szostakowska B, Kruminis-Lozowska W, Racewicz M, Knight R, Tamang L, Myjak P (2004). *Cryptosporidium parvum* and *Giardia lamblia* recovered from flies on a cattle farm and in a landfill. Appl Environ Microbiol.

[38] Lima MSCS, Soares MRA, Pederassi J, Aguiar BCG, Pereira CAS (2014). The housefly *Musca domestica* L. (Diptera: Muscidae) as a potential paratenic host in the city of Bom Jesus-Piauí, Brazil. Comun Sci..

[39] De Oliveira VC, de Mello RP, d'Almeida JM (2002). Muscoid dipterans as helminth eggs mechanical vectors at the zoological garden, Brazil. Rev Saude Publica.

[40] Hemmati S, Afshar AA, Mohammadi MA, Afgar A, Nasibi S, Harandi MF (2018). Experimental and field investigation of non-biting flies as potential mechanical vectors of *Echinococcus granulosus* eggs. Exp Parasitol.

[41] Yu F, Qi M, Zhao Z, Lv C, Wang Y, Wang R (2018). The potential role of synanthropic rodents and flies in the transmission of *Enterocytozoon bieneusi* on a dairy cattle farm in China. J Eukaryot Microbiol.

[42] Pornruseetriratn S, Maipanich W, Sa-nguankiat S, Pubampen S, Poodeepiyasawat A, Thaenkham U (2017). A simple and effective multiplex PCR technique for detecting human pathogenic taenia eggs in houseflies. Southeast Asian J Trop Med Public Health.

[43] Zhao Z, Dong H, Wang R, Zhao W, Chen G, Li S (2014). Genotyping and subtyping *Cryptosporidium parvum* and *Giardia duodenalis* carried by flies on dairy farms in Henan, China. Parasit Vectors.

[44] Sulaiman IM, Fayer R, Bern C, Gilman RH, Trout JM, Schantz PM (2003). Triosephosphate isomerase gene characterization and potential zoonotic transmission of *Giardia duodenalis*. Emerg Infect Dis.

[45] Monzon RB, Sanchez AR, Tadiaman BM, Najos OA, Valencia EG, De Rueda RR (1991). A comparison of the role of *Musca domestica* (Linnaeus) and *Chrysomya megacephala* (Fabricius) as mechanical vectors of helminthic parasites in a typical slum area of Metropolitan Manila. Southeast Asian J Trop Med Public Health.

[46] Barnes AN, Davaasuren A, Baasandavga U, Lantos PM, Gonchigoo B, Gray GC (2021). Zoonotic enteric parasites in Mongolian people, animals, and the environment: using one health to address shared pathogens. PLoS Negl Trop Dis.

[47] Wang ZD, Liu Q, Liu HH, Li S, Zhang L, Zhao YK (2018). Prevalence of *Cryptosporidium*, *Microsporidia* and *Isospora* infection in HIV-infected people: a global systematic review and meta-analysis. Parasit Vectors.

[48] Egger M, Davey Smith G, Schneider M, Minder C (1997). Bias in meta-analysis detected by a simple, graphical test. BMJ.

[49] Graczyk TK, Knight R, Tamang L (2005). Mechanical transmission of human protozoan parasites by insects. Clin Microbiol Rev.

[50] Graczyk TK, Grimes BH, Knight R, Da Silva AJ, Pieniazek NJ, Veal DA (2003). Detection of *Cryptosporidium parvum* and *Giardia lamblia* carried by synanthropic flies by combined fluorescent in situ hybridization and a monoclonal antibody. Am J Trop Med Hyg.

[51] Atiokeng Tatang RJ, Tsila HG, Wabo PJ (2017). Medically important parasites carried by cockroaches in Melong subdivision, Littoral, Cameroon. J Parasitol Res.

[52] Erol U, Danyer E, Sarimehmetoglu HO, Utuk AE (2021). First parasitological data on a wild grey wolf in Turkey with morphological and molecular confirmation of the parasites. Acta Parasitol.

[53] Rondón S, Cavallero S, Renzi E, Link A, González C, D'Amelio S (2021). Parasites of free-ranging and captive American primates: a systematic review. Microorganisms.

[54] Ahmed M, Singh MN, Bera AK, Bandyopadhyay S, Bhattacharya D (2011). Molecular basis for identification of species/isolates of gastrointestinal nematode parasites. Asian Pac J Trop Med.

[55] Lymbery AJ, Thompson RC (2012). The molecular epidemiology of parasite infections: tools and applications. Mol Biochem Parasitol.

[56] Duflot M, Setbon T, Midelet G, Brauge T, Gay M (2021). A review of molecular identification tools for the Opisthorchioidea. J Microbiol Methods.

[57] Xiao L, Escalante L, Yang C, Sulaiman I, Escalante AA, Montali RJ (1999). Phylogenetic analysis of *Cryptosporidium* parasites based on the small-subunit rRNA gene locus. Appl Environ Microbiol.

[58] Essid R, Chelbi H, Siala E, Bensghair I, Menotti J, Bouratbine A (2017). Polymorphism study of *Cryptosporidium hominis* gp60 subtypes circulating in Tunisia. Microb Pathog.

[59] Appelbee AJ, Frederick LM, Heitman TL, Olson ME (2003). Prevalence and genotyping of *Giardia duodenalis* from beef calves in Alberta, Canada. Vet Parasitol.

[60] Lalle M, Pozio E, Capelli G, Bruschi F, Crotti D, Cacciò SM (2005). Genetic heterogeneity at the beta-giardin locus among human and animal isolates of *Giardia duodenalis* and identification of potentially zoonotic subgenotypes. Int J Parasitol.

[61] Cacciò SM, Beck R, Lalle M, Marinculic A, Pozio E (2008). Multilocus genotyping of *Giardia duodenalis* reveals striking differences between assemblages A and B. Int J Parasitol.

[62] Knols B, Smallegange RC (2009). Book review: public health significance of urban pests. Lancet Infect Dis.

[63] Collinet-Adler S, Babji S, Francis M, Kattula D, Premkumar PS, Sarkar R (2015). Environmental factors associated with high fly densities and diarrhea in Vellore. India Appl Environ Microbiol.

[64] Pava-Ripoll M, Pearson RE, Miller AK, Ziobro GC (2012). Prevalence and relative risk of *Cronobacter* spp., *Salmonella* spp., and *Listeria monocytogenes* associated with the body surfaces and guts of individual filth flies. Appl Environ Microbiol..

[65] Chen Y, Qin H, Huang J, Li J, Zhang L (2022). The global prevalence of *Cryptosporidium* in sheep: a systematic review and meta-analysis. Parasitology.

